# What requirements do primary care physicians have with regard to dementia diagnostics and dementia care? – a survey study among general practitioners in Germany 2022/2023

**DOI:** 10.1186/s13690-023-01174-4

**Published:** 2023-08-24

**Authors:** Julian Wangler, Michael Jansky

**Affiliations:** Centre for General Medicine and Geriatrics, University Medical Center of the Johannes Gutenberg, University Mainz, Am Pulverturm 13, 55131 Mainz, Germany

**Keywords:** Dementia care, Dementia diagnosis, Early detection, General practitioner, Attitudes

## Abstract

**Background:**

General practice offers good conditions to detect and provide care for dementia-related diseases. Nonetheless, the effectiveness of dementia care in general practice is repeatedly criticised. To date, few studies have attempted to form a comprehensive picture of the status quo of dementia care in general practice that focuses on GP perspectives of experience and action. The aim of this study was to identify potential strengths and weaknesses of GP-based dementia care, by means of combined consideration of relevant care and treatment dimensions (construct of ‘dementia sensitivity’).

**Methods:**

Through an online poll, a total of 4,511 GPs who are active as treatment providers in Baden-Württemberg, Hesse, Rhineland-Palatinate and Saarland were surveyed between September 2022 and January 2023. In addition to the descriptive analysis, a T-test with independent samples was used to identify significant differences between two groups (interval-scaled or metric variables). Pearson’s chi-squared test (χ2) was used to analyze the percentage values. Two levels of significance were tested for (mean difference at p < 0.05 and p < 0.001). In the course of the analysis, there were particular differences with regard to the sociodemographic variables ‘urban vs. rural doctors’ and ‘doctors with geriatric training vs. doctors with no geriatric training’. Therefore, a complete listing of these parameters is given in the tables. In addition, the factor analysis method was employed.

**Results:**

The respondents consider it important for GPs to provide care and support for dementia patients. The doctors express the desire to offer active support to patients and their family caregivers. At the same time, many GPs experience challenges and difficulties when it comes to practical diagnostic steps (in line with guidelines), the (early) identification of dementia and consistent disease management, including the anticipation of care and treatment needs. Moreover, it appears that a significant proportion of the sample has only limited confidence when it comes to review relevant help and support services. One consistent finding is that some doctors in urban practices who also have geriatric training show substantial increases in knowledge and information with regard to dementia care.

**Conclusions:**

In the light of the findings, it seems particularly advisable to strengthen the geriatric competence of GPs. Moreover, it seems to be essential to ensure that they are better informed about cooperation and support structures in the area of dementia care and better integrated into these.

**Table:** 

**Text box 1. Contributions to the literature**
• To date, there are hardly any studies that have attempted to form a comprehensive picture of the status quo of general-practice dementia care, especially with regard to the perspective of GPs themselves in order to identify strengths and weaknesses in the setting.
• In our results, many GPs find it challenging to offer diagnostic steps with regard to the detection of dementia and disease management, including anticipation of care and treatment needs. It seems important to strengthen the geriatric competence of GPs and to integrate them more into support structures.
• The study has made a contribution to recognized gaps in the literature by revealing conditions and predictors of the effectiveness of general-practice dementia care and approaches to further optimization.

## Background

Currently, approx. 1.8 million people in Germany currently have dementia; this figure could double by 2050 [1]. Primary care by GPs is associated with positive potential in the sensitive detection and care of dementia-related diseases as well as the avoidance of critical care scenarios, which is explained by the good, continuous patient knowledge of general practitioners [2–5]. However, various studies have demonstrated that there are sometimes shortcomings in general-practice care for dementia patients [2, 6–13].

This is initially manifested in relatively low rates of detection and diagnosis [2, 4, 14–17]. Moreover, there are indications of a lack of knowledge of guidelines and treatment options [2, 3, 18, 19], as well as inadequate assessment and exclusion diagnosis [20]. Corresponding with this, several works identified a generally low level of willingness among GPs to employ dementia tests consistently and at an early stage [4, 21]. Moreover, studies show that a significant proportion of GPs tend to leave dementia diagnosis (in line with guidelines) to specialists and favour limited involvement in the care process [9, 22, 23]. One central reason for the current difficulties is that the high level of specialisation required for the detection and care of dementia-related diseases is rarely consistent with the high pressure on time and resources in the setting of general practice and the unselected patient population [10, 12, 24–26].

Despite such findings, few studies have attempted to form a comprehensive picture of the status quo of general practice dementia care while focusing on the GP perspective [9]. The aim of the present study was to identify potential strengths and weaknesses in the setting of general practice by means of combined consideration of relevant care and treatment dimensions with regard to dementia patients and their family caregivers. On this basis, conclusions should be drawn about conditions and predictors of the quality and effectiveness of general-practice dementia care and approaches to further optimisation should be revealed.

The research interest of the study included the following questions:

- Which opinions and attitudes are held by GPs with regard to dementia diagnosis and care?
- Which behaviour patterns do they demonstrate when dealing with dementia patients and their family caregivers?
- To what extent do they experience specific challenges in the care and how substantial are these?
- How do GPs regard their own competence in respect of diagnosis (in line with guidelines) and disease management?
- Which central approaches to the improvement of GP-based dementia care can be derived from the results?

## Methods

### Study design and questionnaire

The study employed a quantitative research design. By means of a written survey, a large number of GPs were to be questioned in order to obtain reliable findings about the aforementioned knowledge interest.

The questionnaire was largely adopted from a sub-project from the cluster-randomised *DemStepCare* study sponsored by the German Innovation Funds, where it was successfully developed, tested and applied in the course of an accompanying evaluation [27–29]. Within this regionally limited care model, only a small number of registered GPs (N = 63) were asked about three different project dates (panel survey) in order to be able to examine possible care effects; there were also questions about the process evaluation. The latter were omitted from the questionnaire that was adapted for the study.

Unlike in the context of *DemStepCare*, the present study was designed as a cross-sectional survey concerned not with examination of positive care effects as the result of a specific intervention but rather with broad recording of the status quo of general-practice dementia care. To this end, a package of indicators of dementia competence and sensitivity was requested in order to establish a comprehensive picture of the situation.

The dementia sensitivity survey undertaken (completion time: 10–12 min, 18 questions) combines indicators of attitudes, actions and competence with respect to dementia diagnosis and care. Based on the preliminary studies conducted in recent years in the context of general practice [incl. 9–11] and with the aid of further studies [inter alia 2, 4, 8, 19, 20, 30, 31], dementia sensitivity has been operationalised as a construct with a focus on subjects including the recording of attitudes to the symptoms of dementia, assumptions of self-efficacy in general practice, relevant indicators for diagnosis, management and compliance with guidelines, capacity to provide care, and perceived challenges.

### Study participants and ethics approval

The survey, which was designed as an online questionnaire, was conducted between September 2022 and January 2023. This was preceded by a written letter in the post, inviting participation by all GPs active as statutory health insurance treatment providers in Baden-Württemberg (6,665), Hesse (3,840), Rhineland-Palatinate (2,669) and Saarland (839).

Participants received no reimbursement of expenses. Consent to participation in the study was given at the start of the online survey.

During this study, no sensitive patient data were gathered and no clinical tests were performed. This was a strictly anonymised survey of a total of 4,511 German GPs. The Ethics Commission of the German federal state of Rhineland-Palatinate informed us that approval by an ethics committee was unnecessary.

### Statistical and data analysis

After cleaning the dataset, the data were analyzed using SPSS 23.0 for Windows. The tables show values including the mean (X̄) and median (˜x). In addition to the descriptive analysis, a T-test with independent samples was used to identify significant differences between two groups (interval-scaled or metric variables). This parametric method has a high test strength and is considered to be statistically robust. The necessary conditions were met with the number of cases, the normal distribution of the groups to be distinguished and the fact that the samples come from the same population [32]. Pearson’s chi-squared test (χ2) was used to analyze the percentage values, since when analyzing frequencies this test should be used [32]. Two levels of significance were tested for (mean difference at p < 0.05 and p < 0.001). In the course of the analysis, there were particular differences with regard to the sociodemographic variables ‘Urban vs. rural doctors’ and ‘Doctors with geriatric training vs. doctors with no geriatric training’. Therefore, a complete listing of these parameters is given in the tables.

To provide a clearer picture of different indicators of attitudes, actions and competence with respect to dementia care on the basis of clusters, the factor analysis method (Varimax rotation) was employed, in which variables are combined into factors on the basis of systematic relationships (correlations) [33]. The conditions for the factor analysis were tested in advance (sampling adequacy according to Kaiser-Meyer-Olkin, significant result in Bartlett’s test of sphericity, commonalities of all the included variables above the threshold of 0.5). The value 0.4/-0.4 was selected as the threshold from which an item is loaded onto a factor.

Answers to free-form questions were analyzed using a subsequent coding based on a qualitative content analysis [34]. As part of reviewing the responses, a category system was developed, which was iteratively assessed and modified as necessary as analysis proceeded. The following sections also present selected quotes given by GPs as answers to the free-form questions.

## Results

### Sample

Of the 4,557 questionnaires processed, 4,511 fully completed forms were included in the analysis (overall response rate: 32%). The sample is structured as follows:

- Gender: 52% male, 48% female.
- Practice environment: 37% large town or city, 63% rural or small town.
- Form of practice: 38% individual practice, 48% group practice, 14% other.
- Patients per quarter: 12% < 1,000, 36% 1,000–1,500, 52% > 1,500.
- Average age: 54 (median: 55).
- Additional training or specialisation in geriatrics: 32%.

### Care intensity

40% of respondents assume that the proportion of older patients (over 65) in their practice is somewhat or substantially above average compared with other practices; 46% assume a roughly average number (15% below average).

58% of respondents state that the practice clientele frequently includes patients with dementia; 33% of such patients come to the practice at least occasionally (10% rarely). Among doctors in small towns and rural communities, 65% state that they frequently have consultations with dementia patients, compared with 48% of doctors in large towns and cities (p < 0.001;Pearson’s χ2 test). 83% of doctors with a geriatric training background state that they frequently look after people with dementia, whereas the proportion is 44% (p < 0.001; Pearson’s χ2 test) among other doctors.

### Diagnosis and interdisciplinary cooperation

When asked about the patients who were found to have or suspected of having (incipient) dementia in recent years, 98% of respondents state that the initial indicators that alerted them to the presence of possible dementia were clues from relatives. 81% mention their own dealings with the patients, whereas 59% cite the complaints of the patients themselves. 37% obtained indications of potential dementia-related disease from the practice staff.

Whereas 86% of respondents state that they use dementia tests in suspected cases or for progress monitoring, 80% employ them as part of geriatric basic assessment. 35% use dementia tests specifically for screening purposes (outside the Basic Geriatric Assessment). It is noticeable that doctors with geriatric training use dementia tests for progress monitoring significantly more frequently than doctors who do not have this background (59% compared with 39%; p < 0.001; Pearson’s χ2 test).

40% of GPs can call on dementia-trained staff in their own practice who have received relevant training. Among doctors with geriatric training, the proportion of trained staff is significantly greater than among doctors who have not received adequate training (49% compared with 34%; p < 0.001; Pearson’s χ2 test). Doctors who have dementia-trained staff state more frequently that they have obtained indications of potential dementia-related disease from the practice staff (48% compared with 29%; p < 0.001; Pearson’s χ2 test).

Only 11% of respondents generally diagnose dementia themselves (in line with S3 guidelines); 69% generally refer patients with suspected dementia for further assessment or diagnosis (20% very varied). 87% of respondents referred the patients to the resident neurologist or psychiatrist; 52% occasionally referred them to a memory clinic.

24% of GPs are generally involved with the treatment of dementia patients and undertake relevant tasks in consultation with specialists. A further 23% provide treatment support only in individual/exceptional cases. 53% generally leave the treatment exclusively to specialists. An open-ended question identified common reasons for GPs to rely heavily on specialists:

*“To be honest, I see myself as referring patients suffering from dementia to specialists. Because we have neither the competence nor the structures here to provide responsible and comprehensive diagnostics and care.”*

*“Dealing with people with dementia is difficult and requires a high level of background knowledge and sensitivity. I see specialists and memory clinics best equipped for this.”*

### Dementia sensitivity

The survey of indicators of dementia sensitivity shows different priorities, strengths and weaknesses of care in general practice (see Table 1). The majority of respondents therefore regard care for this group of patients as a genuine task of GPs, which should not be left solely to specialists. Respondents are also generally aware of the importance of effective and timely dementia diagnosis, especially when this is in compliance with the guidelines. Many respondents regard their own self-efficacy as a given when it comes to contributing to quality of life for people with dementia and their family caregivers. At the same time, noticeable problems are manifested especially in the practical, diagnostic differentiation of dementia-related diseases from other cognitive impairments and the anticipation of care and treatment needs. The respondents also appeared relatively unconfident in respect of consultations that encourage compliance (suspected dementia or dementia diagnosis). Furthermore, many GPs reported that they are not very familiar with help and support services to which patients and their family caregivers can be referred.

**Table 1:** Indicators of dementia sensitivity, rotated component matrix. Question: *Please indicate how strongly you agree with the respective statement.* (0 = “Totally disagree” to 10 = “Totally agree”)

		Rotated component matrix					
	Average (median)	Comp. 1 (explained variation: 25.5%)	Comp. 2 (explained variation: 14.7%)	Comp. 3 (explained variation: 10.9%)	Comp. 4 (explained variation: 8.1%)	Urban vs. rural doctors	Doctors with geriatric training vs. doctors with no geriatric training
“It is often not easy to distinguish the symptoms of dementia from typical signs of ageing (e.g. such as depression).”	X̄: 7.3 (˜x: 8.0)	,223	-,193	,012	,671	X̄: 7.5 (˜x: 8.0) / X̄: 7.1 (˜x: 8.0)	X̄: 7.3 (˜x: 7.0) / X̄: 7.3 (˜x: 7.0)
“As a GP it is important to me to assess suspected dementia with a diagnosis of my own.”	X̄: 6.3 (˜x: 7.0)	-,216	,216	-,193	,036	X̄: 6.3 (˜x: 6.0) / X̄: 6.3 (˜x: 7.0)	X̄: 6.9 (˜x: 7.0) / X̄: 5.9 (˜x: 6.0) *
“Patients with dementia should be treated largely by specialists such as neurologists or psychiatrists, not by their GP.”	X̄: 3.6 (˜x: 3.0)	,037	,019	,302	,678	X̄: 3.7 (˜x: 5.0) / X̄: 3.5 (˜x: 3.0)	X̄: 2.3 (˜x: 3.0) / X̄: 4.4 (˜x: 3.0) *
“A dementia diagnosis does not change much, as the consequence for treatment is inadequate.”	X̄: 2.0 (˜x: 2.0)	-,218	,055	,843	,133	X̄: 1.8 (˜x: 1.0) / X̄: 2.0 (˜x: 2.0)	X̄: 1.7 (˜x: 2.0) / X̄: 2.2 (˜x: 1.0)
“I usually perform dementia tests only if patients or caregivers ask for them.”	X̄: 2.6 (˜x: 3.0)	-,092	,081	,789	,045	X̄: 3.0 (˜x: 3.0) / X̄: 2.3 (˜x: 1.0)	X̄: 2.6 (˜x: 3.0) / X̄: 2.6 (˜x: 3.0)
“Suspected dementia or a dementia diagnosis often results in a high level of defensiveness or tensions with the patient.”	X̄: 5.7 (˜x: 6.0)	,253	-,557	,392	,094	X̄: 6.1 (˜x: 6.0) / X̄: 5.6 (˜x: 6.0)	X̄: 5.7 (˜x: 5.0) / X̄: 5.6 (˜x: 5.0)
“A dementia diagnosis in line with the guidelines is essential for further patient care.”	X̄: 7.6 (˜x: 8.0)	,741	,131	-,070	,039	X̄: 7.7 (˜x: 8.0) / X̄: 7.4 (˜x: 7.5)	X̄: 7.3 (˜x: 8.0) / X̄: 7.7 (˜x: 9.0)
“Early detection of a dementia-related disease is advantageous for the patient.”	X̄: 8.2 (˜x: 9.0)	,831	,095	-,068	,108	X̄: 8.5 (˜x: 8.0) / X̄: 8.1 (˜x: 9.0)	X̄: 8.2 (˜x: 8.0) / X̄: 8.3 (˜x: 8.0)
“If I suspect dementia, I perform a dementia test as a matter of principle.”	X̄: 6.5 (˜x: 7.5)	,319	,559	-,008	-,062	X̄: 7.0 (˜x: 8.0) / X̄: 6.1 (˜x: 7.0)	X̄: 6.5 (˜x: 7.0) / X̄: 6.5 (˜x: 7.0)
“It is challenging to phrase suspected dementia in such a way as to ensure compliance and further patient care.”	X̄: 7.5 (˜x: 8.0)	,858	-,083	,117	-,193	X̄: 7.7 (˜x: 8.5) / X̄: 7.4 (˜x: 8.0)	X̄: 7.4 (˜x: 7.5) / X̄: 7.6 (˜x: 8.0)
“The effect of informing a patient of suspected dementia or a dementia diagnosis is often more adverse than helpful.”	X̄: 2.2 (˜x: 2.0)	-,653	-,197	,315	-,173	X̄: 2.4 (˜x: 2.5) / X̄: 2.3 (˜x: 2.0)	X̄: 2.5 (˜x: 3.0) / X̄: 2.0 (˜x: 2.0)
“It is important to me to support dementia patients and their disease progression in the longer term.”	X̄: 8.4 (˜x: 9.0)	,927	,093	-,035	-,065	X̄: 8.5 (˜x: 8.5) / X̄: 8.3 (˜x: 8.0)	X̄: 8.4 (˜x: 8.0) / X̄: 8.4 (˜x: 8.0)
“I often find it difficult to assess the care needs of dementia patients correctly.”	X̄: 7.5 (˜x: 8.0)	-,101	-,116	-,122	,747	X̄: 7.5 (˜x: 7.0) / X̄: 7.5 (˜x: 7.0)	X̄: 6.2 (˜x: 7.0) / X̄: 8.3 (˜x: 8.0) *
“As a GP there is little that I can do to improve the quality of life of dementia patients.”	X̄: 2.2 (˜x: 2.5)	,038	-,140	,678	-,068	X̄: 2.5 (˜x: 2.5) / X̄: 2.0 (˜x: 2.0)	X̄: 1.5 (˜x: 2.0) / X̄: 2.6 (˜x: 2.0)
“I am very familiar with help and support services for dementia patients and their caregivers.”	X̄: 5.2 (˜x: 5.5)	,094	,796	,118	-,089	X̄: 6.5 (˜x: 6.5) / X̄: 4.7 (˜x: 6.0) *	X̄: 6.2 (˜x: 6.0) / X̄: 4.7 (˜x: 5.0) *
“It is the GP’s job to contribute to better quality of life for caregivers.”	X̄: 7.7 (˜x: 8.0)	,617	,402	,116	,331	X̄: 7.7 (˜x: 8.0) / X̄: 7.7 (˜x: 8.0)	X̄: 7.4 (˜x: 8.0) / X̄: 7.8 (˜x: 8.0)
“If relatives who provide care for dementia patients come to me with questions about organising care, I am easily able to help them.”	X̄: 6.2 (˜x: 7.0)	,291	,870	,055	-,108	X̄: 7.1 (˜x: 7.0) / X̄: 5.6 (˜x: 6.0) *	X̄: 7.0 (˜x: 7.5) / X̄: 5.7 (˜x: 5.0) *
“I make patients and their caregivers aware of specific support and advice services (e.g. dementia networks, care support centres).”	X̄: 6.4 (˜x: 5.0)	,232	,856	,090	-,106	X̄: 7.2 (˜x: 8.0) / X̄: 5.6 (˜x: 6.0) *	X̄: 6.4 (˜x: 7.0) / X̄: 6.4 (˜x: 7.0)
“I find it easy to assess which treatments are appropriate for dementia patients.”	X̄: 3.7 (˜x: 3.0)	-,106	,757	-,160	,054	X̄: 4.3 (˜x: 5.0) / X̄: 3.4 (˜x: 3.0)	X̄: 4.7 (˜x: 5.0) / X̄: 3.0 (˜x: 3.0) *
“I find the existing guidelines for dementia diagnosis and treatment helpful.”	X̄: 6.0 (˜x: 5.0)	,416	,490	-,153	,049	X̄: 7.0 (˜x: 8.0) / X̄: 5.5 (˜x: 5.0) *	X̄: 6.2 (˜x: 6.0) / X̄: 5.8 (˜x: 5.0)
“Dementia can be delayed effectively with the use of medications.”	X̄: 5.2 (˜x: 5.0)	-,040	,283	-,058	,280	X̄: 4.8 (˜x: 5.0) / X̄: 5.4 (˜x: 5.0)	X̄: 5.4 (˜x: 6.0) / X̄: 5.0 (˜x: 5.0)
		Extraction meth.: Principal component analysis Rotation meth.: Varimax, Kaiser normalisation Rotation converged in 6 iterations Explained total variance: 59.2% Sample adequacy according to Kaiser-Meyer-Olkin: 0.59 Significance according to Bartlett: p < 0.001				Significant difference: * p < 0.001; T-test	

Urban doctors assume that they are considerably more familiar with support services in the area of dementia and with referrals to them as required. The same applies for respondents with geriatric training, although these also find it less difficult to anticipate potential care needs.

In respect of attitudes and behaviour patterns with regard to dementia care, factor analysis reveals various clusters of doctors. The largest group considers it vital to ensure continuous general-practice dealings with dementia-related diseases and effective diagnosis. Within the second cluster, one of the focuses is on advice and referrals for patients and their family caregivers. The third and fourth clusters are clearly dominated by sceptical and resigned attitudes with regard to diagnosis, care and communication.

The survey of subjective competence assessments confirms a lack of confidence among many GPs with regard to the process of diagnosis in line with the guidelines and appropriate disease management (see Table 2). It is noticeable that the personal sense of competence is significantly weaker among doctors in small towns and rural communities compared with doctors in large towns and cities. The self-assessments of respondents with geriatric training are similarly positive.

**Table 2:** Subjective competence assessment in the process of diagnosis and treatment

Question (0 = “Not at all good” to 10 = “Excellent”)	Average (median)	Urban vs. rural doctors	Doctors with geriatric training vs. doctors with no geriatric training
*In general, how would you rate your knowledge/competence with respect to the diagnosis process for dementia-related diseases?*	X̄: 5.7 (˜x: 6.0)	X̄: 6.5 (˜x: 6.0) / X̄: 5.2 (˜x: 6.0) *	X̄: 6.5 (˜x: 7.0) / X̄: 5.0 (˜x: 5.0) *
*In general, how would you rate your knowledge/competence with respect to the treatment process for and management of dementia-related diseases?*	X̄: 5.4 (˜x: 6.0)	X̄: 6.5 (˜x: 7.0) / X̄: 4.8 (˜x: 5.0) *	X̄: 6.3 (˜x: 7.0) / X̄: 4.9 (˜x: 5.0) *
*What would you say? How familiar are you with the S3 guidelines for dementia diagnosis and treatment?*	X̄: 4.1 (˜x: 4.0)	X̄: 5.1 (˜x: 6.0) / X̄: 3.5 (˜x: 3.0) *	X̄: 5.8 (˜x: 6.0) / X̄: 3.1 (˜x: 2.0) *
*How confident do you feel when comes to making a dementia diagnosis in line with the guidelines? (This means the S3 guidelines.)*	X̄: 4.0 (˜x: 4.0)	X̄: 5.0 (˜x: 4.0) / X̄: 3.5 (˜x: 3.0) *	X̄: 5.3 (˜x: 5.0) / X̄: 3.3 (˜x: 3.0) *
*How confident do you feel when comes to managing a dementia-related disease in line with the guidelines, i.e. supporting with treatment and medication? (This means the S3 guidelines.)*	X̄: 4.4 (˜x: 5.0)	X̄: 5.3 (˜x: 7.0) / X̄: 3.8 (˜x: 4.0) *	X̄: 5.7 (˜x: 6.0) / X̄: 3.4 (˜x: 3.5) *
	Significant difference: * p < 0.001; T-test		

### Challenges in practice and optimization approaches

With the aid of a set of items, the GPs were explicitly asked to what extent they find various aspects and activity processes of dementia care challenging in everyday practice (see Table 3). Alongside successful treatment management, differential diagnosis clarification is considered especially difficult, as are also communication and compliance problems that can arise in the patient consultation when explaining the diagnosis.

**Table 3:** Challenges experienced in dementia diagnosis and care. Question: Please state respectively how great you consider the following challenges to be, on the basis of your personal experience. (0 = “Very low” to 10 = “Very great”)

		Rotated component matrix				
	Average (median)	Comp. 1 (explained variation: 55.3%)	Comp. 2 (explained variation: 11.9%)	Comp. 3 (explained variation: 8.0%)	Urban vs. rural doctors	Doctors with geriatric training vs. doctors with no geriatric training
Application of the dementia guidelines (S3)	X̄: 5.8 (˜x: 6.5)	0.207	0.133	0.898	X̄: 5.8 (˜x: 5.0) / X̄: 5.8 (˜x: 6.0)	X̄: 5.4 (˜x: 5.0) / X̄: 6.1 (˜x: 6.0)
Steps towards suspected dementia and further action	X̄: 5.5 (˜x: 5.5)	0.128	0.647	0.222	X̄: 4.7 (˜x: 5.0) / X̄: 5.6 (˜x: 6.0) *	X̄: 4.7 (˜x: 4.0) / X̄: 5.5 (˜x: 6.0) *
Diagnosis and testing process (completion of the diagnosis process and interpretation)	X̄: 5.2 (˜x: 5.0)	0.463	0.712	0.056	X̄: 4.4 (˜x: 5.0) / X̄: 5.7 (˜x: 6.0) *	X̄: 5.2 (˜x: 5.0) / X̄: 5.2 (˜x: 5.0)
Differentiation of dementia from other forms of cognitive impairment (e.g. depression)	X̄: 6.7 (˜x: 7.0)	0.220	0.806	0.159	X̄: 6.2 (˜x: 6.0) / X̄: 6.8 (˜x: 7.0)	X̄: 5.9 (˜x: 6.0) / X̄: 7.0 (˜x: 7.0) *
Gaining the acceptance and cooperation of the patient for the performance of dementia tests	X̄: 6.6 (˜x: 7.0)	0.246	0.826	0.020	X̄: 5.8 (˜x: 5.0) / X̄: 6.6 (˜x: 7.0) *	X̄: 6.3 (˜x: 6.0) / X̄: 6.8 (˜x: 7.0)
Explaining the diagnosis to the patient and ensuring compliance	X̄: 6.2 (˜x: 7.0)	0.684	0.440	0.228	X̄: 6.2 (˜x: 6.0) / X̄: 6.2 (˜x: 6.0)	X̄: 6.1 (˜x: 7.0) / X̄: 6.3 (˜x: 6.0)
Successful treatment support for dementia patients	X̄: 6.8 (˜x: 7.0)	0.883	0.219	0.107	X̄: 6.8 (˜x: 7.0) / X̄: 6.8 (˜x: 7.0)	X̄: 6.3 (˜x: 7.0) / X̄: 7.0 (˜x: 7.0)
Consultation with dementia patients	X̄: 6.6 (˜x: 6.5)	0.937	0.115	0.151	X̄: 6.5 (˜x: 7.0) / X̄: 6.7 (˜x: 7.0)	X̄: 6.1 (˜x: 6.0) / X̄: 6.9 (˜x: 8.0)
Communication with relatives who provide care	X̄: 6.1 (˜x: 6.0)	0.792	0.287	0.120	X̄: 6.0 (˜x: 6.0) / X̄: 6.1 (˜x: 6.0)	X̄: 5.5 (˜x: 6.0) / X̄: 6.6 (˜x: 7.0) *
Advising dementia patients and their relatives on help and support services	X̄: 5.5 (˜x: 6.0)	0.707	0.446	0.348	X̄: 4.8 (˜x: 5.0) / X̄: 5.9 (˜x: 6.0) *	X̄: 5.1 (˜x: 6.0) / X̄: 5.8 (˜x: 5.0)
Advising dementia patients and their relatives on legal aspects (welfare power of attorney, care, ‘driving’)	X̄: 6.3 (˜x: 7.0)	0.241	0.583	0.528	X̄: 5.4 (˜x: 5.5) / X̄: 6.8 (˜x: 7.0) *	X̄: 5.6 (˜x: 5.0) / X̄: 6.7 (˜x: 7.0) *
		Extraction meth.: Principal component analysis Rotation meth.: Varimax, Kaiser normalisation Rotation converged in 5 iterations Explained total variance: 75.2% Sample adequacy according to Kaiser-Meyer-Olkin: 0.88 Significance according to Bartlett: p < 0.001				
		Significant difference: * p < 0.001; T-test				

In line with the findings above, doctors in the urban setting and with a geriatric training background find certain problems less concerning, such as diagnostic procedures or dealing with and advising dementia patients and their family caregivers.

Factor analysis reveals three groups of GPs. The first group finds challenges principally in communication contexts; the other two in diagnosis and treatment. In all clusters, a high proportion of doctors find that advising patients and their family caregivers is demanding.

An open-ended question asked after the quantitative determination confirmed these results. Below are some examples of statements that have been made widely by the respondents:

Diagnosing dementia is not automatically the way to help the patient. It is (much more) important to keep the quality of life high for the patient and their caregivers and to make life with dementia possible with the help of certain support services. This is exactly where I often feel overwhelmed.

The diagnostic clarification and differentiation from other diseases poses an enormous challenge for the possibilities that GPs have.

It is not easy to make the patient understand that he or she is affected by dementia and to set the course for this to be accepted. From my point of view, this communicative hurdle remains significant in everyday practice.

In order to improve the identification and care of patients suffering from dementia, the GPs advocate better payment for dementia-related counseling, diagnosis and care services (78%). In view of the challenges of performing effective differential diagnostics under conditions of daily time and cost pressure, 80% consider the introduction of a structured, evidence-based diagnostic and therapeutic algorithm to be rather or very helpful. Just as many respondents (82%) think that increased collaboration between GPs and regional support networks would be helpful. However, some of these would have to be created first. 71% plead for increased training of practice staff with regard to early detection and diagnostics as well as support with healthcare services. This applies, for example, to the overview of the regional dementia care structures: Only every second GP believes to be familiar with the offers available in the area.

## Discussion

### Main findings

General practice offers good conditions to detect and provide continuous care for dementia-related diseases. The current lack of relevant research was seen as a reason to take a holistic approach to dementia sensitivity of GPs, as part of a large-scale survey. This examined the indicators of attitudes, actions and competence with respect to dementia care in general practice.

As the results show, the respondents consider it important for GPs to be present on matters to do with the care of dementia patients and for them to have the confidence to contribute to improving the quality of life of those affected and their family caregivers. Most GPs express the desire not only to refer patients and their relatives to specialists but also to offer them active support.

At the same time, it is clear that many GPs find it challenging to offer practical diagnostic steps (in line with guidelines) in the identification of dementia and consistent disease management, including anticipation of the care and treatment needs of patients and their family caregivers in time-sensitive everyday practice. Moreover, it appears that a significant proportion of the sample has only limited confidence to review relevant help and support services and to make referrals to them as required.

One consistent finding is that some doctors in urban practices who also have geriatric training show substantial increases in knowledge and information with regard to dementia care. This is especially evident when it comes to the overview of local or regional help and support services in the area of dementia and referrals to the same. In addition, doctors with geriatric training are more frequently able to call on trained practice staff.

### Comparison with previous studies

The results are reflected in the findings of other studies, according to which there is a range of obstacles in the care reality of general practice [2, 7–10, 12, 35]. Low et al. identified that the decision of GPs to use consistent dementia diagnosis and to undertake relevant tasks in disease management is dependent on factors including attitudes and opinions with regard to the symptoms and available treatment options, personal confidence with regard to diagnosis and communication skills, the psychosocial resources available to patients and their relatives, as well as medical knowledge and the availability of supporting stakeholders [6].

Even in our preliminary studies [9–11] a large proportion of GPs involved stated that differential diagnosis clarification was challenging under the conditions of time and cost pressure. Lack of confidence in the differentiation of dementia from other forms of cognitive impairment is amplified by lack of clarity in the process of diagnosis and treatment as well as by problems in cooperation with specialists. Internationally too, systematic reviews have been presented, which state that there is “a lack of training and confidence” on the part of primary medical carers in the area of dementia care, amplified by systemic barriers and especially “a lack of time during consultations and lack of support services” [7]. Additionally, there are signs of a lack of communication confidence when making a dementia diagnosis, as well as indications that GPs may not know enough about non-pharmacologic interventions [6, 9, 16, 30].

The tendency of urban doctors to have a better overview of care services can be explained by the fact that such services are more available and more differentiated in the urban setting [13, 14, 36, 37]. Taking a closer look at additional geriatric competence seems to be more substantiated in this respect and can, according to the survey results, make a considerable difference to the medical handling of dementia-related diseases. In other studies, too, it was noticeable that GPs with no geriatric training perform fewer dementia screenings compared with colleagues who have received further training, more rarely have trained practice staff, and give less consideration to indications from practice staff in the detection of dementia [10, 11, 22]. The same applies for knowledge and systematic application of the S3 guidelines [19, 30, 38]. Not least, geriatric background knowledge can help with the successful implementation of stabilising consultation strategies [39, 40]. In the light of the findings, it seems advisable to strengthen the geriatric competence of GPs.

Earlier studies and systematic reviews also found that many GPs are not sufficiently familiar with regional care structures for dementia patients and their family caregivers. Mansfield et al. as well as Low et al. state that the lack of available or actively consulted support networks in the area of dementia care can be a limiting factor for medical care [6, 7, 23]. For Germany, one key reason for these shortcomings is the widespread lack of multi-professional structures for outpatient crisis intervention, linking up medical, nursing and other stakeholders [17, 23, 27, 28]. In conjunction with this, it is frequently impossible to achieve systematic and timely classification of care needs and risks. This gap in care is increasingly leading to crisis situations in care and complication-ridden hospitalisations [41, 42]. Consequently, it seems essential to introduce GPs more to cooperation and support structures in the area of dementia care [7, 23, 27, 43, 44]. This means not only sound knowledge of support services but also stronger cooperation with regional stakeholders. Timely referral of patients and their relatives to regional advice and care networks [9] can both enhance patient care and reduce the risk of ‘burnout’ for family caregivers[38].

### Strengths and limitations

Although it was possible to obtain a large, heterogeneous sample of primary care providers, various limitations of the survey must be reflected. These include a regional recruitment focus in two federal states and a limited response. Moreover, it is possible that GPs with an interest in the subject may have shown stronger participation in the survey (selection bias). This is suggested by the very high proportion of doctors with geriatric training.

This study was only a cross-sectional study and only correlative relationships can be uncovered, but – due to the lack of longitudinal data – not robust causal relationships. For example, it is not clear whether GPs who have become increasingly involved with support services for people with dementia in recent years have recognized the need for geriatric continuing education and then completed it, or that participation in geriatric continuing education has led to these physicians becoming more familiar with the relevant support services. Another limitation is that the analysis was not controlled for multiple testing. Therefore, the results of the study can only be seen as hypothesis-generating or explorative, which must be validated again in another study [45].

## Conclusions

As the results show, the respondents consider it important for GPs to be present on matters to do with the care of dementia patients. Most doctors express the desire to offer active support to patients and their relatives. At the same time, it is clear that many GPs find it challenging to offer practical diagnostic steps (in line with guidelines) in the identification of dementia and consistent disease management, including anticipation of care and treatment needs in time-sensitive everyday practice. Moreover, it appears that a significant proportion of the sample has only limited confidence to review relevant help and support services and to make referrals to them as required.

In the light of the findings, it seems advisable to strengthen the geriatric competence of GPs. Moreover, it seems to be essential to provide general practitioners with a stronger explanation of cooperation and support structures in the area of dementia care and to integrate them more into these.

## Data Availability

All major data generated or analyzed during this study are included in this published article. Additional information can be provided on request made to the corresponding author.

## References

[1] World Health Organization. Global status report on the public health response to dementia. WHO; 2021.

[2] Pentzek M, Vollmar HC, Wilm S (2017). Putting dementia awareness into general practice: the CADIF approach. Z Gerontol Geriatr.

[3] Tilburgs B, Vernooij-Dassen M, Koopmans R et al. Barriers and facilitators for GPs in dementia advance care planning: A systematic integrative review. PLoS One 2018; 13 (6): e0198535 PMID: 29924837 [10.1371/journal.pone.0198535]. 10.1371/journal.pone.0198535 PMC6010277 29924837

[4] Thyrian JR, Hertel J, Wucherer D (2017). Effectiveness and safety of dementia Care Management in Primary Care: a randomized clinical trial. JAMA Psychiatry.

[5] Yaffe MJ, Orzeck P, Barylak L (2008). Family physicians’ perspectives on care of dementia patients and family caregivers. Can Fam Physician.

[6] Low L-F, McGrath M, Swaffer K (2019). Communicating a diagnosis of dementia: a systematic mixed studies review of attitudes and practices of health practitioners. Dementia.

[7] Mansfield E, Noble N, Sanson-Fisher R (2019). Primary Care Physicians’ perceived barriers to optimal dementia care: a systematic review. Gerontologist.

[8] Pentzek M, Wollny A, Wiese B (2009). Apart from nihilism and stigma: what influences general practitioners’ accuracy in identifying incident dementia?. Am J Geriatr Psychiatry.

[9] Wangler J, Jansky M. Dementia Diagnostics in General Practitioner Care. Do General Practitioners Have Reservations? - The Findings of a Qualitative Study in Germany. Wien Med Wochenschr 2020; 170 (9–10): 230–237. PMID: 31811446 [10.1007/s10354-019-00722-4]. 10.1007/s10354-019-00722-4 PMC7272384 31811446

[10] Wangler J, Jansky M (2021). Factors influencing general practitioners’ perception of and attitude towards dementia diagnostics and care – results of a survey among primary care physicians. Wien Med Wochenschr.

[11] Wangler J, Jansky M (2022). Slowing dementia symptoms - a qualitative study on attitudes and experiences of general practitioners in Germany. Eur J Gen Pract.

[12] Wollny A, Fuchs A, in der Schmitten J (2010). Between Closeness and Distance: family practitioners’ perception of persons with dementia. Z Allg Med.

[13] Petrazzuoli F, Vinker S, Koskela TH (2017). Exploring dementia management attitudes in primary care: a key informant survey to primary care physicians in 25 european countries. Int Psychogeriatr.

[14] Connolly A, Gaehl E, Martin H (2011). Underdiagnosis of dementia in primary care: variations in the observed prevalence and comparisons to the expected prevalence. Aging Ment Health.

[15] Eichler T, Thyrian JR, Hertel J (2014). Rates of formal diagnosis in people screened positive for dementia in primary care: results of the DelpHi-Trial. J Alzheimers Dis.

[16] Löppönen M, Raiha I, Isoaho R et al. Diagnosing cognitive impairment and dementia in primary health care – a more active approach is needed. Age Ageing 2003; 32 (6): 606–612. PMID: 14600001 [10.1093/ageing/afg097]. 10.1093/ageing/afg097 14600001

[17] Jennings AA, Foley T, Walsh KA et al. General practitioners’ knowledge, attitudes, and experiences of managing behavioural and psychological symptoms of dementia: A mixed methods systematic review. Int J Geriatr Psychiatry 2018; ; 33 (9): 1163–1176. PMID: 29900592 [10.1002/gps.4918]. 10.1002/gps.4918 PMC6099359 29900592

[18] Pimlott NJ, Persaud M, Drummond N (2009). Family physicians and dementia in Canada: part 1. Clinical practice guidelines: awareness, attitudes, and opinions. Can Fam Physician.

[19] Dreier-Wolfgramm A, Michalowsky B, Austrom MG et al. Dementia care management in primary care: Current collaborative care models and the case for interprofessional education. Z Gerontol Geriatr 2017; 50 (Suppl2): 68–77. PMID: 28364258 [10.1007/s00391-017-1220-8]. 10.1007/s00391-017-1220-8 28364258

[20] Thyrian JR, Fiss T, Dreier A et al. Life- and person-centred help in Mecklenburg-Western Pomerania, Germany (DelpHi): study protocol for a randomised controlled trial. Trials 2012; 13 (1): 56. PMID: 22575023 [10.1186/1745-6215-13-56]. 10.1186/1745-6215-13-56 PMC3482148 22575023

[21] Iliffe S, Manthorpe J, Eden A. Sooner or later? Issues in the early diagnosis of dementia in general practice. Family Practice 2003; 20 (4): 376–381. PMID: 12876106 [10.1093/fampra/cmg407]. 10.1093/fampra/cmg407 12876106

[22] Donath C, Gräßel E, Großfeld-Schmitz M et al. Effects of general practitioner training and family support services on the care of home-dwelling dementia patients – Results of a controlled cluster-randomized study. BMC Health Serv Res 2010; 10: 314. PMID: 21087474 [10.1186/1472-6963-10-314]. 10.1186/1472-6963-10-314 PMC2996386 21087474

[23] Virnaua L, Braesigkb A, Deutsch T et al. GPs’ willingness to participate in research networks in Germany. Scand J Prim Health Care 2022; 40 (2): 237–245. PMID: 35770652 [10.1080/02813432.2022.2074052]. 10.1080/02813432.2022.2074052 PMC9397419 35770652

[24] Thoonsen B, Vissers K, Verhagen S et al. Training general practitioners in early identification and anticipatory palliative care planning: a randomized controlled trial. BMC Fam Pract 2015; 16: 126. PMID:26395257 [doi:10.1186/s12875-015-0342-6]. 10.1186/s12875-015-0342-6 PMC4578268 26395257

[25] Van Hout H, Vernooij-Dassen M, Bakker K et al. General practitioners on dementia: tasks, practices and obstacles. Patient Educ Couns 2000; 39 (2–3): 219–225. PMID: 11040721 [10.1016/s0738-3991(99)00034-8]. 10.1016/s0738-3991(99)00034-8 11040721

[26] Teel CS. Rural practitioners’ experiences in dementia diagnosis and treatment. Aging and Mental Health 2004; 8 (5): 422–429. PMID: 15511740 [10.1080/13607860410001725018]. 10.1080/13607860410001725018 15511740

[27] Bablok I, Binder H, Graf E, Stelzer D et al. Primary dementia care based on the individual needs of the patient: Study protocol of the cluster randomised controlled study DemStepCare. BMC Geriatr 2021; 21 (1): 222. PMID:33794789 [doi:10.1186/s12877-021-02114-z]. 10.1186/s12877-021-02114-z PMC8012747 33794789

[28] Khanassov V, Vedel I. Family physician-case manager collaboration and needs of patients with dementia and their caregivers: a systematic mixed studies review. Ann Fam Med 2016; 14 (2): 166–77. PMID: 26951593 [10.1370/afm.1898]. 10.1370/afm.1898 PMC4781521 26951593

[29] Gemeinsamer Bundesausschuss Innovationsausschuss [Federal Joint Committee, Innovation Committee]. : DemStepCare – Hausarztbasierte Demenzversorgung mit koordinierter Kooperation und risikostratifiziertem Einsatz spezialisierter Pflegekräfte [Dementia care from general practitioners with coordinated cooperation and risk-ratified use of specialised care]. [https://innovationsfonds.g-ba.de/projekte/neue-versorgungsformen/demstepcare-hausarztbasierte-demenzversorgung-mit-koordinierter-kooperation-und-risikostratifiziertem-einsatz-spezialisierter-pflegekraefte.279] Accessed on 25 May 2023.

[30] Tang EYH, Birdi R, Robinson L. Attitudes to diagnosis and management in dementia care: views of future general practitioners. Int Psychogeriatr 2018; 30 (3): 425–430. PMID: 27502828 [10.1017/S1041610216001204]. 10.1017/S1041610216001204 27502828

[31] Connell CM, Boise L, Stuckey JC (2004). Attitudes toward the diagnosis and disclosure of dementia among family caregivers and primary care physicians. Gerontologist.

[32] Bortz J, Schuster C (2010). Statistik für Human- und Sozialwissenschaftler [Statistics for human and social scientists].

[33] Fromm S, Baur N, Fromm S (2008). Faktorenanalyse. Datenanalyse mit SPSS für fortgeschrittene [Data analysis with SPSS for advanced users, german].

[34] Mayring P. Qualitative Inhaltsanalyse. Grundlagen und Techniken [Qualitative content analysis. Basics and Techniques]. 11. Aufl. Weinheim: Beltz, 2010.

[35] Eichler K, Scrabal C, Steurer J et al. Preventive health risk appraisal for older people and impact on GP’s patient management: a prospective study. Family Practice 2007; 24 (6): 604–609. PMID: 17986626 [10.1093/fampra/cmm063]. 10.1093/fampra/cmm063 17986626

[36] Fletcher B, Gheorghe A, Moore D et al. Improving the recruitment activity of clinicians in randomised controlled trials: a systematic review. BMJ Open 2012; 2 (1): e000496. PMID: 22228729 [10.1136/bmjopen-2011-000496]. 10.1136/bmjopen-2011-000496 PMC3253423 22228729

[37] Riedel O, Klotsche J, Wittchen H-U (2016). Overlooking Informal Dementia Caregivers’ Burden. Res Gerontol Nurs.

[38] Whitlatch CJ, Orsulic-Jeras S. Meeting the informationducational, and psychosocial support needs of persons living with dementia and their family caregivers. Gerontologist. 2018; 58 (Suppl 1): 58–73. PMID: 29361068 [10.1093/geront/gnx162]. 10.1093/geront/gnx162 29361068

[39] Thyrian JR, Eichler T, Michalowsky B et al. Community-dwelling people screened positive for dementia in primary care: A comprehensive, multivariate descriptive analysis using data from the DelpHi-study. J Alzheimers Dis 2016; 52 (2): 609–617. PMID: 27031481 [10.3233/JAD-151076]. 10.3233/JAD-151076 27031481

[40] Tilburgs B, Koopmans R, Vernooij-Dassen M (2020). Educating Dutch General Practitioners in Dementia Advance Care Planning: a Cluster Randomized Controlled Trial. J Am Med Dir Assoc.

[41] Lehmann J, Michalowsky B, Kaczynski A et al. The Impact of Hospitalization on Readmission, Institutionalization, and Mortality of People with Dementia: A Systematic Review and Meta-Analysis. J Alzheimers Dis 2018; 64 (3): 735–749. PMID: 29966191 [10.3233/JAD-171128]. 10.3233/JAD-171128 29966191

[42] Wolf D, Rhein C, Geschke K et al. Preventable hospitalizations among older patients with cognitive impairments and dementia. Int Psychogeriatr 2019; 31 (3): 383–391. PMID: 30221613 [10.1017/S1041610218000960]. 10.1017/S1041610218000960 30221613

[43] Tilburgs B, Koopmans R, Schers H et al. Advance care planning with people with dementia: a process evaluation of an educational intervention for general practitioners. BMC Fam Pract 2020; 21 (1): 199. PMID: 32967619 [10.1186/s12875-020-01265-z]. 10.1186/s12875-020-01265-z PMC7513545 32967619

[44] Winblad B, Amouyel P, Andrieu S (2016). Defeating Alzheimer’s disease and other dementias: a priority for european science and society. Lancet Neurol.

[45] Bender R, Lange S, Ziegler A. Multiple testing. Dtsch Med Wochenschr 2007; 132 (Suppl 1): 26–29. PMID: 17530590 [10.1055/s-2007-959035]. 10.1055/s-2007-959035 17530590

